# Assessing the effectiveness of statin therapy for alleviating cerebral small vessel disease progression in people ≥75 years of age

**DOI:** 10.1186/s12877-020-01682-w

**Published:** 2020-08-17

**Authors:** Yuqi Guo, Yunpeng Li, Xukui Liu, Yi Cui, Yingxin Zhao, Shangwen Sun, Qing Jia, Qiang Chai, Gary Gong, Hua Zhang, Zhendong Liu

**Affiliations:** 1 Basic Medical College, Shandong First Medical University, Jinan, 250062 Shandong China; 2 Key Laboratory of Rare and Uncommon Diseases, Basic Medical Colleg, Shandong First Medical University, Jinan, 250062 Shandong China; 3 School of Medicine and Life Sciences, Shandong First Medical University, Zhangqiu, 250202 Shandong China; 4 Department of Radiology, Shandong University Qilu Hospital, Jinan, 250012 Shandong China; 5 Cardio-Cerebrovascular Control and Research Center, Basic Medical Colleg, Shandong First Medical University, No. 18877, Jingshi Road, Jinan, 250062 Shandong China; 6 The Russel H. Morgan Department of Radiology and Radiological Sciences, the Johns Hopkins University School of Medicine, Baltimore, MD 21287 USA

**Keywords:** Statins, Neuroprotection, Cerebral small vessel disease, Aging

## Abstract

**Background:**

Statins have been recommended by several guidelines as the primary prevention medication for cardiovascular diseases. However, the benefits of statin therapy for cerebral small vessel disease (CSVD), particularly in adults ≥75 years of age, have not been fully evaluated.

**Methods:**

We analyzed the data from a prospective population-based cohort study and a randomized, double-blind, placebo-controlled clinical trial to determine whether statin therapy might aid in slowing the progression of CSVD in adults ≥75 years of age. For the cohort study, 827 participants were considered eligible and were included in the baseline analysis. Subsequently, 781 participants were included in follow-up analysis. For the clinical trial, 227 participants were considered eligible and were used in the baseline and follow-up analyses.

**Results:**

The white matter hyperintensities (WMH) volume, the WMH-to-intracranial volume (ICV) ratio, the prevalence of a Fazekas scale score ≥ 2, lacunes, enlarged perivascular spaces (EPVS), and microbleeds were significantly lower in the statin group than the non-statin group at baseline in the cohort study (all *P* < 0.05). During the follow-up period, in both the cohort and clinical trial studies, the WMH volume and WMH-to-ICV ratio were significantly lower in the statin/rosuvastatin group than the non-statin/placebo group (all *P* < 0.001). Statin therapy was associated with lower risk of WMH, lacunes, and EPVS progression than the non-statin therapy group after adjustment for confounders (all *P* < 0.05). There was no statistically significant difference in the risk of microbleeds between the statin and non-statin therapy groups (all, *P* > 0.05).

**Conclusions:**

Our findings indicated that statin therapy alleviated the progression of WMH, lacunes, and EPVS without elevating the risk of microbleeds. On the basis of the observed results, we concluded that statin therapy is an efficient and safe intervention for CSVD in adults ≥75 years of age.

**Trial registration:**

Chictr.org.cn: ChiCTR-IOR-17013557, date of trial retrospective registration November 27, 2017 and ChiCTR-EOC-017013598, date of trial retrospective registration November 29, 2017.

## Background

Cerebral small vessel disease (CSVD) is an age-related clinical cognitive syndrome encompassing a group of pathological processes with multifarious etiologies that affect the small vessels in the brain [1–4]. Some prominent features associated with CSVD include white matter hyperintensities (WMH), lacunes, enlarged perivascular space (EPVS), and microbleeds observed through magnetic resonance imaging (MRI) [5, 6]. CSVD is responsible for 25–30% of all cerebral strokes and as much as 45% of all dementia [1, 7]; it is also frequently associated with mood disorders and gait problems [1, 7–9]. CSVD poses a high burden on individuals and society worldwide. Therefore, measures to prevent and treat CSVD are particularly and increasingly important.

CSVD is strongly associated with cardiovascular risk factors including hypertension, hyperlipidemia, and aging [1, 9–11]. However, the results of the Rotterdam Scan Study have demonstrated that blood pressure is not associated with WMH progression and incident lacunar infarcts in the oldest people [12]. Moreover, the interaction between blood pressure and age in relation to WMH progression and incident lacunar infarcts is not statistically significant [12]. Blood pressure in the progression of CSVD may differ with age, and antihypertensive therapy may not ameliorate the progression of CSVD in the oldest people. Therefore, does lipid-lowering treatment slow the progression of CSVD in the oldest people?

Statins competitively inhibit hydroxy methyl glutaryl coenzyme A reductase and hence have been recommended by several guidelines as the medication for primary prevention of cardiovascular diseases [13–15]. The association between statin therapy and CSVD is well documented in middle aged (40 to 65 years of age) and older (66 to 75 years of age) adults [16]. A recent meta-analysis [17] has indicated that statins produce significant decreases in major vascular events irrespective of age. Other studies [7–9, 18] have demonstrated that statins are independent beneficial factors alleviating the progression of CSVD. However, there is less direct evidence of the benefits of statin therapy for slowing the progression of CSVD among people older than 75 years because the adverse effect risks of statins, such as frailty, liver injury, and myalgias are more serious in adults ≥75 than < 75 years of age [17].

To elucidate whether statin therapy is beneficial in slowing the progression of CSVD in adults ≥75 years of age, we extracted and analyzed the data of participants ≥75 years of age in our two previous studies. One study was a prospective population-based cohort study with participants ≥15 years of age [11], and the other was a randomized, double-blind, placebo-controlled clinical trial with participants ≥60 years of age [7, 9]. Our main goal was to investigate the effect of statins on the progression of CSVD in adults 75 years and older.

## Methods

### Study population

The participants in this study were from a prospective cohort study (study ID: ChiCTR-EOC-17013598) [11] and a randomized clinical trial (study ID: ChiCTR-IOR-17013557) [7, 9], which have been described in detail elsewhere. For the cohort study, briefly, 21,000 participants ≥15 years of age who had no plans to leave the area within 5 years and had not been included in other studies were enrolled from community dwellings through multistage and cluster sampling in the Shandong area, China, between 2007 and 2009. Among them, 827 participants ≥75-years-old with statins as the primary prevention method were eligible and included in this study. The exclusion criteria were as follows: dementia, Alzheimer’s disease, Parkinson’s disease, schizophrenia seizures, history of stroke, congestive heart failure, acute myocardial infarction in the previous 6 months, liver disease, renal failure and dialysis, malignancy, drug or alcohol abuse, contraindications to MRI, and unwillingness to provide informed consent. For the randomized clinical trial, participants were hypertensive patients ≥60 years of age who were recruited between April 2008 and November 2010 from the Shandong area, China. Hypertension was defined as a systolic/diastolic blood pressure ≥ 140/90 mmHg, a previous diagnosis of hypertension, or current use of antihypertensive medication. A total of 251 patients ≥75 years of age were included in this study according to the following exclusion criteria: secondary hypertension, definite hypersensitivity, or contraindication to the study medications, and the exclusion criteria for the cohort study.

The randomized clinical trial in this study was conducted in compliance with the Declaration of Helsinki and adhered to the Good Clinical practice guidelines. Ethical approval for the two studies including this study were obtained from the Research Ethics Committee of the Institute of Basic Medicine, Shandong Academy of Medical Sciences. Each participant provided written informed consent.

### Study design and follow-up

The design and follow-up of this study have been described elsewhere. The cohort study was a prospective, longitudinal, and observational study [11]. Participants were followed up every 6 months after the baseline clinical visit. The demographic and clinical characteristics of each participant were collected at every clinical visit. The assessment of statin use was performed with a questionnaire including details such as the name of statine, dose, compliance (months per year), and adverse effects. For participants with hypertension or diabetes, blood pressure or glucose-lowering medications were recommended by public health physicians if participants were willing to receive treatment. According to the observational design, there was no unified medication schedule for participants in this study. CSVD was determined with brain MRI at the baseline (2008–2009), first MRI follow-up (2010–2012), and second MRI follow-up (2013–2015) visits. In this study, participants were dichotomized to a non-statin group (< 6 months/year) and statin group (≥6 months/year) on the basis of the assessment of statin use, because few participants (3.2%) used statins for 0–6 months/year and delayed the clinical benefits of statins [19].

For the clinical trial [7, 9], patients were separately randomly assigned in a 1:1:1:1 ratio into blood pressure lowering intervention and lipid-modulating intervention arms after a 2-week washout period in the original study. The determination of sample size and the details of randomization methods in the original study have been described elsewhere [7, 9]. Among all patients, 124 patients ≥75 years of age were assigned to a placebo group, and 127 were assigned to a rosuvastatin (10 mg given once daily) group. Clinical visits in the washout period were conducted weekly. Baseline visits occurred at the end of the washout period, and the follow-up visits were performed at trial months 1, 3, and 6, and thereafter at 6 month intervals. Brain MRI scans were conducted at the baseline (2008–2010), the first brain MRI follow-up assessment (2012–2013), and the second brain MRI follow-up assessment (2015–2017).

### Brain MRI scan

CSVD was determined in accordance with STandards for ReportIng Vascular changes on nEuroimaging (STRIVE) [5, 6] with a 3.0-T Siemens Allegra scanner (Erlangen, Germany) or 3.0-T GE Signa Horizon scanner (General Electric Medical Systems, Milwaukee, WI). The same brain MRI protocols were applied for two studies and every MRI assessment visit. The sequences of MRI scans were as follows: T1-weighted 3D magnetization-prepared rapid gradient echo [repetition time (TR)/echo time (TE) = 1900/3 ms and slice thickness = 1 mm], T2-weighted 3D fast spin-echo (TR/TE = 3000/98 ms and slice thickness = 3 mm), T2*-weighted gradient-echo type echoplanar (TR/RE = 600/16 ms and slice thickness = 3 mm), and fluid-attenuated inversion recovery (FLAIR; TR/TE = 5000/355 ms and slice thickness = 2 mm) sequences [7, 9, 11]. The total WMH volume was computed automatically on FLAIR images from periventricular and subcortical segmentation and corrected by total intracranial volume (ICV). The Fazekas scale of WMH was also assessed on FLAIR images (scores of 0–3 was given for no lesions, punctuate, early confluent lesions, or confluent lesions, respectively) [20]. Lacunes were defined as cavities with diameters of 3–15 mm with cerebrospinal-fluid-like signal intensity on a combination of T1-weighted, T2-weighted, and FLAIR images. EPVS was defined as visible fluid-filled spaces adjacent to cerebral vessels on T2-weighted and FLAIR images and was discriminated from small lacunes of presumed vascular origin [5, 21]. Microbleeds were defined as oval or round, hypointense, and homogeneous foci in the brain parenchyma with diameters 2–10 mm on T2*-weighted images. Calcifications, sulcal vessels, and signal averaging from bone were systematically excluded.

The spatial transformation matrices were obtained after the MRIs were normalized with Montreal Neurological Institute templates [22]. During the normalization, the differences between the MRIs of each participant were corrected with the International Consortium for Brain Mapping template for East Asian Brains. A Gaussian filter was used to smooth the images and to minimize the variability in local anatomy among subjects. All scans were visually inspected for mis-registration errors. The scans were excluded if excessive motion artifacts were found.

Each available scan was independently rated by experienced neuroradiologists who were blinded to all clinical data. Consensus meetings were held if there were disagreements among raters. Limited inter- and intra-rater reliability testing in random samples of 120 patients showed good reliability with the weighted Cohen’s kappa values of 0.86 and 0.86 for the presence of Fazekas scale lesions, 0.81 and 0.80 for lacunes, 0.76 and 0.77 for EPVS, and 0.81 and 0.81 for microbleeds.

### Outcomes

In this study, the primary outcomes included the progression of WMH, lacunes, EPVS, and microbleeds. The progression of WMH was defined as an increase in the WMH volume and WMH-to-ICV ratio, as well as a lower Fazekas scale grade developing to a higher grade. The progression of lacunes, EPVS, and microbleeds was defined by the occurrence of one or more newly diagnosed lacunes, EPVS, and microbleeds during the follow-up duration. Stroke was regarded as the secondary outcome.

### Statistical analysis

The sample size was calculated, although this study included a cohort study and a randomized clinical trial. The calculation formula *n* = 2*σ*^2^ × *f*(*α*, *β*)/(*μ*1-*μ*2) ^2^ [23] was used, where *n* is the sample size of each group, *σ* is the standard deviation (SD) of the baseline WMH volume, *μ*1 is the baseline WMH volume, and *μ*2 is the desired WMH volume at the end of the study. In this study, *α* was equal to 0.05, and *β* was equal to 0.1. On the basis of previous studies [7, 9, 11], the mean and SD of the baseline WMH volume in adults ≥75 years of age were 5.4 and 3.5 mL, respectively, and the mean progressed by approximately 2.0 mL over 60 months. In this study, 807 participants were included in the non-statin/placebo group, and 247 were included in the statin/rosuvastatin group to achieve 85% power with a level of statistical significance of 0.05.

Statistical analyses were conducted in SPSS for Windows (version 24.0; SPSS Inc., Chicago, IL, USA). Continuous data are presented as mean ± SD or median with interquartile range (IQR; the range between the 25th and 75th percentiles) depending on the normality of the data, and categorical data are expressed as a frequency with percentages. We used the Kolmogorov-Smirnov test to determine the normality of continuous data. To assess the difference between groups, Student’s *t*-test or the Mann-Whitney U test was used for continuous data, and the chi-square test was used for categorical data. We applied a linear mixed model to compare the changes in WMH volumes and the WMH-to-ICV ratio across the follow-up period between groups. To assess whether statin therapy was independently associated with CSVD, first we used a multiple linear backward stepwise regression analysis to evaluate the association between statin therapy and the WMH volume and WMH-to-ICV ratio, and a logistic regression model to evaluate the association between statin therapy and the prevalence of a Fazekas scale score ≥ 2, lacunes, EPVS, and microbleeds at baseline in the cohort study. Then we used the Kaplan-Meier with log-rank test to estimate the differences in the cumulative risks of the progression of WMH, lacunes, EPVS, and microbleeds during the follow-up period between groups in the cohort study and the clinical trial. The hazard ratio (HR) with 95% confidence interval (CI) was determined with the Cox proportional hazards model. All models were adjusted for age, sex, smoking, alcohol consumption, history of hypertension and diabetes, medication for hypertension and diabetes, ischemic heart disease, peripheral artery disease, baseline body mass index, systolic and diastolic blood pressure, fasting plasma glucose, triglycerides, high-density lipoprotein cholesterol (HDL-C), and low-density lipoprotein cholesterol (LDL-C). In addition, the baseline WMH-to-ICV ratio, lacunes, EPVS, and microbleeds, the mean and SD of systolic and diastolic blood pressure within the follow-up duration, and incident stroke were included as confounders in the linear mixed model and Cox proportional hazards model. A two-sided *P* value less than 0.05 was considered statistically significant.

### Role of the funding source

The funders had no role in planning the study design, data collection, data analysis and interpretation, or report writing and reviewing. The corresponding author and data analyst had full access to all data in the study and had final responsibility in the decision to submit for publication.

## Results

### Baseline demographic and clinical characteristics

**Fig. 1:**
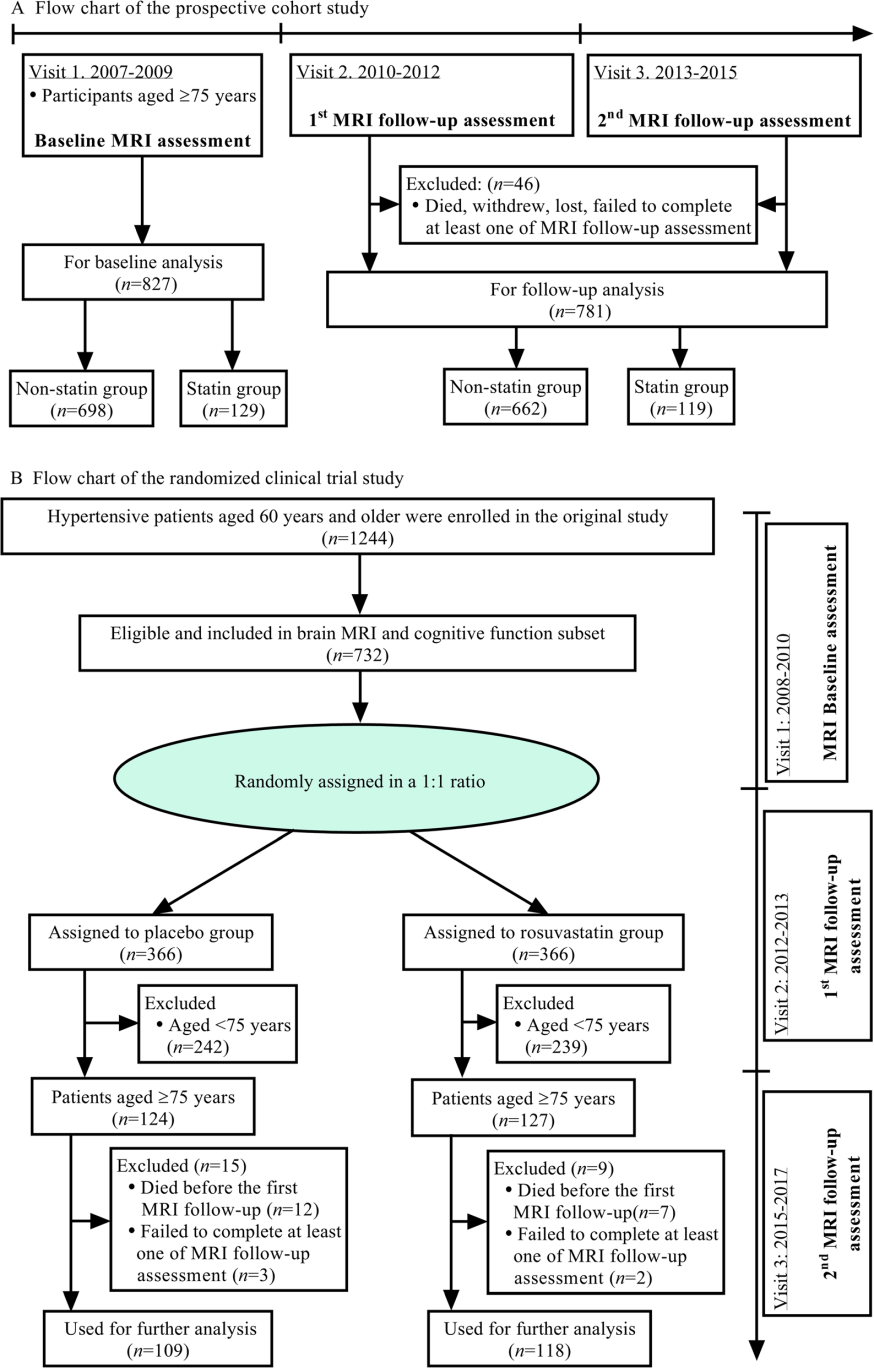
The protocol flow chart

The protocol flowchart of this study is summarized in Fig. 1. For the cohort study, 827 participants were included in the baseline analysis. There were 129 statin users and 698 non-statin users. The statin group treatments included simvastatin, atorvastatin, lovastatin, fluvastatin, pravastatin, and rosuvastatin. The details of the demographic and clinical characteristics of patients are shown in Table 1. As expected, the levels of plasma total cholesterol, triglycerides, and LDL-C were significantly higher, and the level of HDL-C was lower, in the non-statin group than the statin group (all *P* < 0.05). Table 2 shows the details of the baseline demographic and clinical characteristics of the clinical trial. After an average of 63.0 (IQR: 60.0 to 63.0) months of follow-up, 24 hypertensive patients were excluded during the follow-up period (eTable 1), and 227 patients were included in the baseline and follow-up analyses. There were no significant difference in demographic and clinical characteristics between groups (all, *P* > 0.05).

**Table 1:** Demographic and clinical characteristics of the participants aged ≥75 years in the cohortstudy

	All (*n* = 827)	Non-statin group (*n* = 698)	Statin group (*n* = 129)	*P* value^a^
Clinical parameters				
Female (*n* [%])	465 (56.2)	392 (56.2)	73 (56.6)	0.928
Age (years)	78.08 ± 2.38	78.00 ± 2.38	78.50 ± 2.39	0.028
Current smoking (*n* [%])	257 (31.1)	218 (31.2)	39 (30.2)	0.822
Alcohol consumption (*n* [%])	271 (32.8)	232 (33.2)	39 (30.2)	0.504
Hypertension (*n* [%])	554 (67.0)	472 (67.6)	82 (63.6)	0.368
Antihypertensive medication (*n* [%])	425 (51.4)	367 (52.6)	58 (45.0)	0.112
Diabetes (*n* [%])	98 (11.9)	79 (11.3)	19 (14.7)	0.271
Lowering glucose medication (*n* [%])	91 (11.0)	73 (10.5)	18 (14.0)	0.244
Dyslipidemia (*n* [%])	586 (70.9)	490 (70.2)	96 (74.4)	0.333
Ischemic heart disease (*n* [%])	506 (61.2)	422 (60.5)	84 (65.1)	0.319
Peripheral artery disease (*n* [%])	114 (13.8)	93 (13.3)	21 (16.3)	0.371
Need statins for primary prevention (*n* [%])	720 (87.1)	591 (84.7)	129 (100.0)	–
Statins (*n* [%])	129 (15.6)	0 (0.0)	129 (100.0)	–
Simvastatin	46 (5.6)	0 (0.0)	46 (35.7)	–
Atorvastatin	16 (1.9)	0 (0.0)	16 (12.4)	–
Lovastatin	7 (0.8)	0 (0.0)	7 (5.4)	–
Fluvastatin	11 (1.3)	0 (0.0)	11 (8.5)	–
Pravastatin	13 (1.6)	0 (0.0)	13 (10.1)	–
Rosuvastatin	36 (4.4)	0 (0.0)	36 (27.9)	–
Antiplatelet medication (*n* [%])	187 (22.6)	155 (22.2)	32 (24.8)	0.517
Body mass index (kg/m^2^)	24.38 ± 2.53	24.36 ± 2.55	24.52 ± 2.39	0.506
Heart rate (bpm)	70.67 ± 8.64	70.71 ± 8.73	70.48 ± 8.12	0.783
SBP (mm Hg)	147.92 ± 16.69	148.20 ± 16.52	146.36 ± 17.58	0.250
DBP (mm Hg)	75.82 ± 8.29	75.93 ± 8.24	75.21 ± 8.59	0.366
Biochemical parameters				
TCHO (mmol/L)	4.68 ± 0.75	4.72 ± 0.76	4.49 ± 0.68	0.001
TG (mmol/L)	1.53 ± 0.49	1.56 ± 0.51	1.37 ± 0.30	< 0.001
HDL-C (mmol/L)	1.16 ± 0.35	1.15 ± 0.35	1.25 ± 0.35	0.002
LDL-C (mmol/L)	2.82 ± 0.68	2.86 ± 0.68	2.62 ± 0.63	< 0.001
FPG (mmol/L)	5.42 ± 1.31	5.44 ± 1.32	5.30 ± 1.22	0.260
Brain magnetic resonance imaging				
WMH (mL)	5.02 (3.53, 6.37)	5.18 (3.73, 6.51)	4.25 (2.82, 5.48)	< 0.001
WMH-to-ICV ratio (%)	0.38 (0.27, 0.48)	0.40 (0.29, 0.49)	0.32 (0.22, 0.40)	< 0.001
Prevalence of Fazekas scale ≥2 (*n* [%])	114 (13.8)	104 (14.9)	10 (7.8)	0.031
Prevalence of lacunes (*n* [%])	98 (11.9)	90 (12.9)	8 (6.2)	0.031
Prevalence of Virchow-Robin spaces (*n* [%])	140 (16.9)	126 (18.1)	14 (10.9)	0.045
Prevalence of microbleeds (*n* [%])	67 (8.1)	56 (8.0)	11 (8.5)	0.847

Data are expressed as mean ± standard deviation, median with interquartile range, or numbers with percentages. ^a^Indicates the differences between non-statin and statin groups. Abbreviation list: SBP, systolic blood pressure; DBP, diastolic blood pressure; TCHO, total cholesterol; TG, triglycerides; HDL-C, high-density lipoprotein cholesterol; LDL-C, low-density lipoprotein cholesterol; FPG, fasting plasma glucose; WMH, white matter hyperintensities; ICV, intracranial volume

**Table 2:** Demographic and clinical characteristics of the hypertensive patients aged ≥75 years in the clinical trial study

	Placebo group (*n* = 109)	Rosuvastatin group (*n* = 118)	*P* value
Clinical parameters			
Female (*n* [%])	52 (47.7)	57 (48.3)	0.928
Age (years)	78.48 ± 2.58	78.24 ± 2.35	0.465
Current smoking (*n* [%])	28 (25.7)	23 (19.5)	0.264
Alcohol consumption (*n* [%])	34 (31.2)	33 (28.0)	0.594
Body mass index (kg/m^2^)	23.57 ± 2.21	23.94 ± 2.49	0.226
Heart rate (bpm)	67.21 ± 6.41	67.51 ± 5.45	0.706
SBP (mm Hg)	157.23 ± 9.50	156.80 ± 9.74	0.735
DBP (mm Hg)	71.14 ± 7.21	70.76 ± 7.35	0.699
Biochemical parameters			
TCHO (mmol/L)	5.08 ± 0.66	5.12 ± 0.69	0.696
TG (mmol/L)	1.51 ± 0.39	1.48 ± 0.39	0.625
HDL-C (mmol/L)	1.12 ± 0.19	1.17 ± 0.19	0.061
LDL-C (mmol/L)	3.28 ± 0.72	3.28 ± 0.75	0.989
FPG (mmol/L)	5.70 ± 0.66	5.62 ± 0.67	0.333
Brain magnetic resonance imaging			
WMH (mL)	6.57 (5.16, 8.11)	6.93 (4.87, 8.03)	0.907
WMH-to-ICV ratio (%)	0.54 (0.40, 0.69)	0.54 (0.40, 0.66)	0.871
Prevalence of Fazekas scale ≥2 (*n* [%])	27 (24.8)	21 (17.8)	0.199
Prevalence of lacunes (*n* [%])	16 (14.7)	13 (11.0)	0.409
Prevalence of Virchow-Robin spaces (*n* [%])	12 (11.0)	11 (9.3)	0.674
Prevalence of microbleeds (*n* [%])	13 (11.9)	9 (7.6)	0.274

Data are expressed as mean ± standard deviation, median with interquartile range, or numbers with percentages. Abbreviation list: SBP indicates systolic blood pressure; DBP, diastolic blood pressure; TCHO, total cholesterol; TG, triglycerides; HDL-C, high-density lipoprotein cholesterol; LDL-C, low-density lipoprotein cholesterol; FPG, fasting plasma glucose; WMH, white matter hyperintensities; ICV, intracranial volume

### The contribution of statin therapy to CSVD in the cohort study at baseline

We first assessed the correlation between statin therapy and the incidence of CSVD at baseline in the cohort study. The WMH volume, WMH-to-ICV ratio, prevalence of a Fazekas scale score ≥ 2, lacunes, EPVS, and microbleeds were significantly lower in the statin group than the non-statin group (all *P* < 0.05, Table 1). Statin therapy was independently and negatively correlated with WMH volume (beta = − 0.716, 95% CI: − 1.068 to − 0.364, *P* < 0.001) and the WMH-to-ICV ratio (beta = − 0.057, 95% CI: − 0.084 to − 0.031, *P* < 0.001) after adjustment for confounders including age, sex, smoking, alcohol consumption, history of hypertension and diabetes, medication for hypertension and diabetes, baseline body mass index, blood pressure, fasting plasma glucose, and lipids. The risks of a Fazekas scale score ≥ 2, lacunes, and EPVS were significantly lower in the statin group than the non-statin group after adjustment for confounders (all adjusted *P* < 0.05, Table 3). There were no significant differences in the risk of microbleeds between groups (*P* > 0.05, Table 3).

**Table 3:** Correlation between statin therapy and the prevalence of CSVD at baseline in the cohort study

	Beta	Wald	OR (95% CI)	*P* value
Prevalence of Fazekas scale ≥2				
Model 1	−0.783	4.833	0.457 (0.227, 0.919)	0.028
Model 2	−0.734	4.478	0.480 (0.243, 0.947)	0.034
Model 3	−0.734	4.501	0.480 (0.244, 0.946)	0.034
Prevalence of lacunes				
Model 1	−0.917	5.533	0.400 (0.186, 0.858)	0.019
Model 2	−0.842	4.820	0.431 (0.203, 0.914)	0.028
Model 3	−0.806	4.449	0.447 (0.211, 0.945)	0.035
Prevalence ofVirchow-Robin spaces				
Model 1	−0.614	4.305	0.541 (0.296, 0.990)	0.038
Model 2	−0.592	3.916	0.553 (0.307, 0.994)	0.045
Model 3	−0.578	3.581	0.561 (0.315, 0.999)	0.048
Prevalence of microbleeds				
Model 1	0.066	0.037	1.069 (0.544, 2.100)	0.847
Model 2	0.059	0.027	1.060 (0.528, 2.218)	0.869
Model 3	0.017	0.002	1.017 (0.516, 2.007)	0.960

Model 1: Adjusted for age and sex

Model 2: model 1 + smoking, alcohol intake, history of hypertension, diabetes, and dyslipidemia; medication for hypertension, diabetes, and platelet aggregation

Model 3: model 2 + body mass index, blood pressure, fasting blood glucose, and blood lipids

### The contribution of statin therapy to the progression of CSVD in the cohort study over the follow-up period

**Fig. 2:**
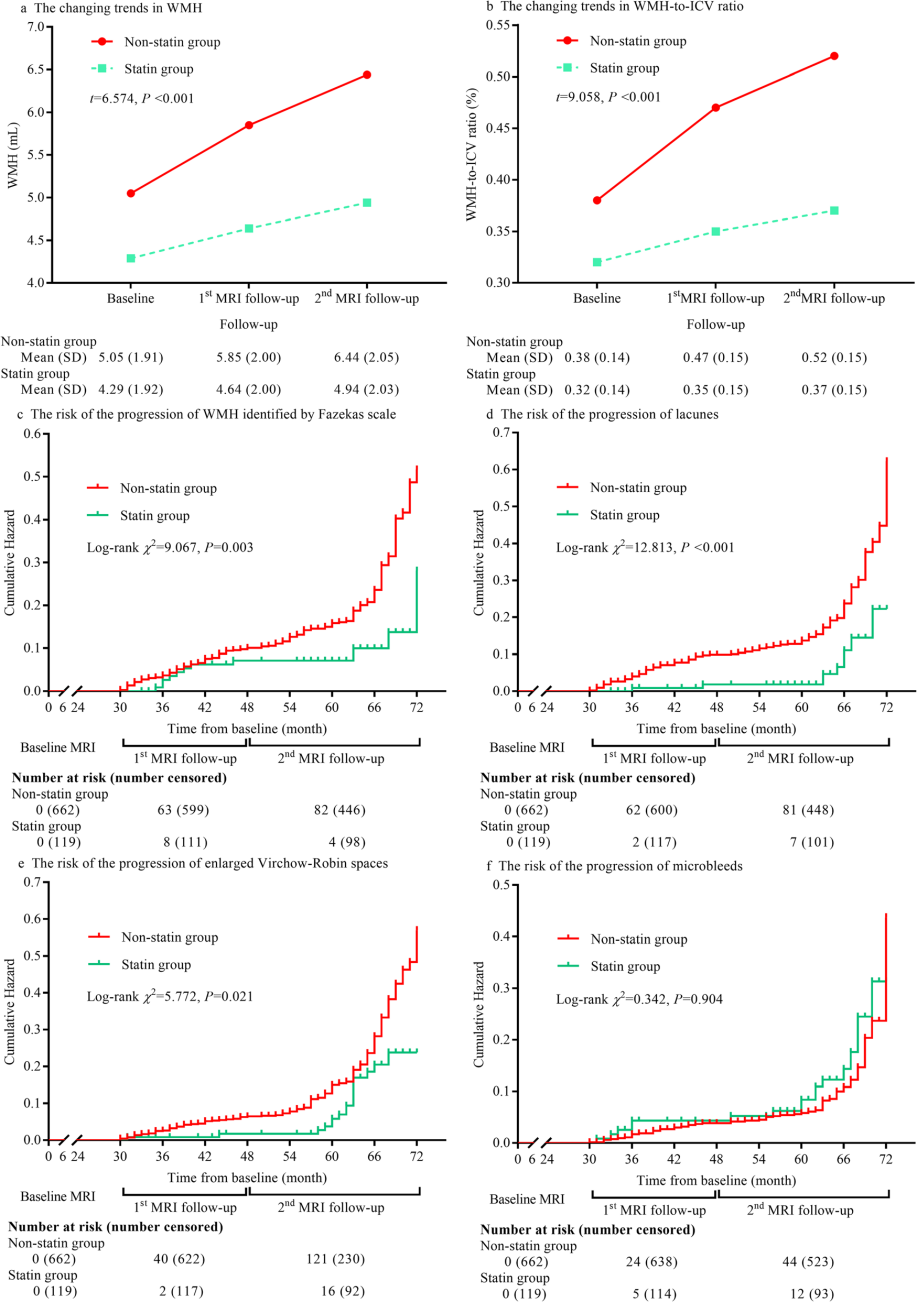
Effect of statins on the progression of cerebral small vessel disease in the cohort study. **a** The trends of changes in WMH volume; **b** the trends of changes in WMH-to-ICV ratio; **c** the cumulative hazard of the risk of WMH progression identified by the Fazekas scale; **d** the cumulative hazard of the risk of lacune progression; **e** the cumulative hazard of the risk of EPVS progression; **f** the cumulative hazard of the risk of microbleed progression. Abbreviations list: WMH, white matter hyperintensities; ICV, intracranial volume; EPVS, enlarged perivascular space

Then we evaluated the association between statin therapy and the risk of CSVD progression in the cohort study. After an average of 63.0 (IQR: 57.0 to 66.0) months of follow-up, 46 patients were excluded because they died, withdrew, were lost to follow-up, or did not complete at least one brain MRI follow-up assessment in the cohort study (eTable 2). Finally, 781 participants were included in the follow-up analysis. Increasing trends in the WMH volume and WMH-to-ICV ratio were observed in the statin and the non-statin groups from baseline to the first and to the second brain MRI follow-up assessments. The increasing trends in WMH volume and WMH-to-ICV ratio were significantly lower in the statin group than the non-statin group (all *P* < 0.001, Fig. 2). Among 781 participants, 157 (20.1%) exhibited WMH progression identified by a lower Fazekas scale grade that later developed to a higher grade, 152 patients (19.5%) exhibited lacune progression, 179 patients (22.9%) exhibited EPVS progression, and 85 patients (10.9%) exhibited microbleed progression. The risks of the progression of WMH (HR: 0.517, 95% CI: 0.338 to 0.791), lacunes (HR: 0.446, 95% CI: 0.290 to 0.686), and EPVS (HR: 0.608, 95% CI: 0.409 to 0.905) were significantly lower in the statin group than the non-statin group. The significant differences remained even after adjustment for confounders including the mean and SD of systolic and diastolic blood pressure within the follow-up duration, and incident stroke (adjusted *P* = 0.003 for WMH progression, *P* < 0.001 for lacune progression, and *P* = 0.021 for EPVS progression, Fig. 2). The mean and SD of systolic and diastolic blood pressures within the follow-up duration are detailed in eTable 3. There was no statistical difference in the risk of microbleed progression between the statin and the non-statin groups (HR: 1.327, 95% CI: 0.741 to 2.379, *P* = 0.904, Fig. 2).

### The contribution of rosuvastatin use to the progression of CSVD in the clinical trial over the follow-up period

**Fig. 3:**
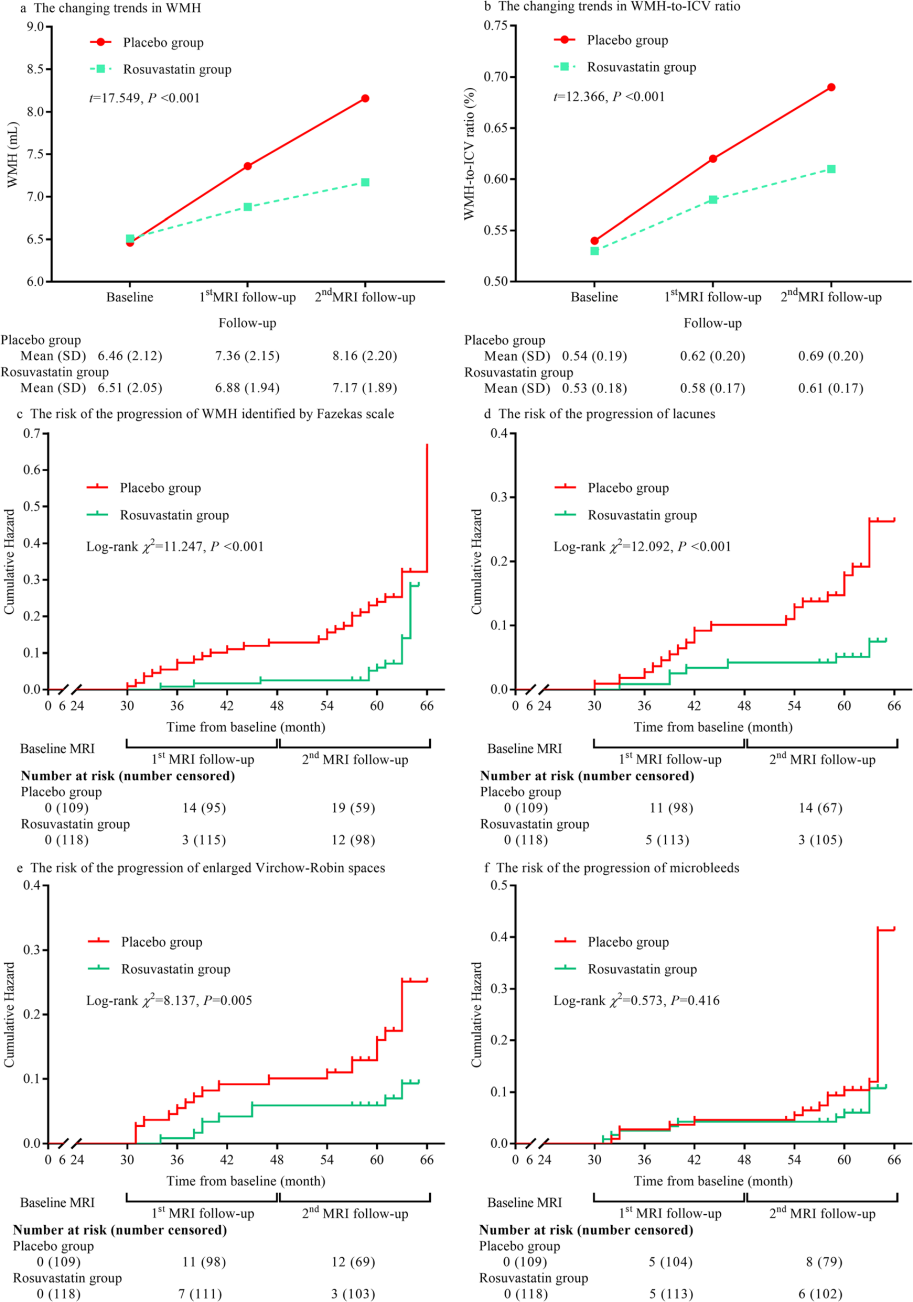
Effect of rosuvastatin on the progression of cerebral small vessel disease in the clinical trial. **a** The trends of changes in WMH volume; **b** the trends of changes in WMH-to-ICV ratio; **c** the cumulative hazard of the risk of WMH progression identified by the Fazekas scale; **d** the cumulative hazard of the risk of lacune progression; **e** the cumulative hazard of the risk of EPVS progression; **f** the cumulative hazard of the risk of microbleed progression. Abbreviations list: WMH, white matter hyperintensities; ICV, intracranial volume; EPVS, enlarged perivascular space

Third, we appraised the association between rosuvastatin use and the risk of CSVD progression in the clinical trial. The trends of changes in WMH volume and WMH-to-ICV ratio in the clinical trial were more similar to those in the cohort study. These trends were significantly lower in rosuvastatin group than the placebo group in the follow-up duration (all, *P* < 0.001, Fig. 3). Among 227 hypertensive patients ≥75 years of age, 48 (21.1%) manifested WMH progression, 33 (14.5%) manifested lacune progression, 33 (14.5%) manifested EPVS progression, and 24 (10.6%) manifested microbleed progression. The risks of progression of WMH (HR: 0.408, 95% CI: 0.233 to 0.716, *P* < 0.001), lacunes (HR: 0.417, 95% CI: 0.257 to 0.676, *P* < 0.001), and EPVS (HR: 0.466, 95% CI: 0.249 to 0.873, *P* = 0.005) were significantly lower in the rosuvastatin group than the placebo group after adjustment for confounders including the mean and SD of systolic and diastolic blood pressure within the follow-up duration, and incident stroke. The mean and SD of systolic and diastolic blood pressure within the follow-up duration are detailed in eTable 3. Furthermore, we observed no statistical difference in microbleed progression between the rosuvastatin and placebo groups (HR: 0.703, 95% CI: 0.374 to 1.692, *P* = 0.416, Fig. 3).

### Incident stroke over the follow-up period

We also assessed the effect of statins on incident stroke. For the cohort study, incident stroke was 64 participants (8.2%) and the risk of incident stroke was significantly lower in the statin group than the non-statin group (HR: 0.598, 95% CI: 0.368 to 0.973, *P* < 0.001). For the clinical trial, the incident stroke was 13 participants (5.7%), and the risk of incident stroke was significantly lower in the rosuvastatin group than the placebo group (HR: 0.560, 95% CI: 0.374 to 0.838, *P* < 0.001).

### Adverse effects of statins

We also assessed the adverse effects of statins. There were no statistically significant differences in the incidence of newly diagnosed diabetes, myalgias, impaired liver function, and frailty between the statin and non-statin groups in the cohort study, and between the rosuvastatin and placebo groups in the clinical trial, over the follow-up duration (all *P* > 0.05, eTable 4).

## Discussion

The goal of this study was to assess the neuroprotective effects of statins in adults ≥75 years of age on the basis of the data generated from a cohort study and a clinical trial. After analysis, we found that (1) statin therapy markedly ameliorated the risks of WMH, lacunes, and EPVS progression; and (2) statin therapy had neither beneficial nor detrimental effects on microbleed progression.

Statin therapy is well established to be beneficial in the primary and secondary prevention of many mortality associated cardio- and cerebrovascular diseases [24–29]. Recently, a large meta-analysis including 28 randomized control trials has demonstrated that statins significantly decrease major vascular events such as heart attack and stroke, irrespective of age [17].

The Regression of Cerebral Artery Stenosis study has indicated that simvastatin ameliorates cerebral WMH progression and is associated with a lower incidence of lacunes in people 36 and 75 years of age [30, 31]. Our previous studies have shown that rosuvastatin intervention markedly decreases the risk of WMH progression and the incidence of new lacunes in hypertensive patients 60 years and older [7, 9]. These results indeed indicated that statins had neuroprotective effects. However, there was less direct evidence of these neuroprotective effects in adults ≥75 years of age.

In this study, we first performed a baseline cross-sectional analysis of the cohort study and then conducted a prospective analysis to validate the findings of the cross-sectional analysis. Finally, we used a randomized, double-blind, placebo-controlled clinical trial to further validate the above findings. We found that statin therapy decreased the risk of progression of WMH, lacunes, and EPVS by almost 50% in the cohort study. To confirm these findings, we analyzed the data from the randomized, double-blind, placebo-controlled clinical trial. The decreases in the risks of WMH, lacunes, and EPVS progression were approximately 64, 72, and 64%, respectively, in the rosuvastatin treated group, as compared with the placebo group, after adjustment for confounders. The results indicated that statin therapy, compared with non-statin therapy, was associated with a lower risk of WMH, lacunes, and EPVS progression.

Statins have been found to be independently associated with a higher risk of cerebral microbleeds [32, 33]. The Stroke Prevention by Aggressive Reduction in Cholesterol Levels (SPARCL) study, conducted in a group of patients with ischemic stroke, has shown that statins, compared with placebo, caused an approximately two-fold increase risk of hemorrhagic stroke [32]. Haussen and colleagues have reported that the presence and increased number of microbleeds was 2.7-fold higher in statin-treated patients than in statin-untreated patients with intracerebral hemorrhage. The differences were more significant in cortico-subcortical microbleeds [33]. In contrast, the results of this study were not consistent with those of our previous studies [7, 9]. We did not find any significant difference in the risk of microbleed progression between groups receiving statin and non-statin therapy. The different participants recruited in these studies were probably a major contributor to the discrepancy. In the SPARCL study, the participants were patients with ischemic stroke, and in Haussen’s study, the participants were patients with intracerebral hemorrhage.

We found that the effects of statin therapy on the progression of WMH, lacunes, and EPVS differed from the progression of microbleeds, possibly because of the different underlying etiologies and pathophysiological mechanisms in CSVD subtypes. WMH, lacunes, and EPVS are considered subtypes of ischemic damage resulting from obstruction or narrowing of cerebral small vessels [34–36]. Microbleeds, however, are regarded as a subtype of hemorrhagic damage caused by red blood cell leakage from brain capillaries and/or bleeding vessels [35, 37]. Microbleeds are also seen as an asymptomatic precursor of intracerebral hemorrhage [33, 35] and have been reported to be associated with the serum lipid [33, 38].

The major strength of this study is that this work included a prospective and population-based cohort study and a randomized, double-blind, and placebo-controlled clinical trial. The results of both studies corroborated each other, thus enhancing the credibility of the results and conclusions. Another strength was that long-term follow-up continued for an average of 63.0 (57.0 to 66.0) months in the cohort study and 63.0 (IQR: 60.0 to 63.0) months in the clinical trial.

Several limitations in our study must be addressed. First, we did not show the changes in blood lipids, particularly LDL-C, in this study. Statins are mainly used to lower blood LDL-C in clinical practice, and a higher level of LDL-C is significantly associated with an increased risk of cardio- and cerebrovascular diseases. Second, we did not include incident mild cognitive impairment or dementia as outcomes in this study. However, CSVD is well documented to be closely associated with mild cognitive impairment and dementia. Third, a surrogate endpoint, WMH reduction, was used in this study but was not a clinical endpoint. Fourth, only rosuvastatin was used in the clinical trial. Although rosuvastatin is an important member of the statin family, it does not fully represent the other statins. In addition, there were fewer statin users than non-statin users in the cohort study, and the participants were merely hypertensive patients in the clinical trial. Therefore bias might have been introduced in the results. Finally, this study was a post-hoc subgroup analysis, a design well recognized to create a risk of type I error.

## Conclusions

In conclusion, we found that statin therapy alleviated the progression of WMH, lacunes, and EPVS without elevating the risk of cerebral microbleeds in adults ≥75 years of age. Our findings indicated that statin therapy was an efficient and safe intervention for CSVD. Beyond the already existing evidence of the potential benefits of statin therapy, we observed neuroprotective effects in adults ≥75 years of age. However, our findings should be validated by further studies including multi-racial and multi-ethnic trials.

## Supplementary information

**Additional file 1: eTable 1.** Demographic and clinical characteristics of the excluded participants ≥75 years of age in the cohort study. **eTable 2.** Demographic and clinical characteristics of the excluded hypertensive patients ≥75 years of age in the clinical trial. **eTable 3.** Mean and variability in blood pressure in the cohort study and clinical trial over the follow-up period. **eTable 4.** Adverse effects of statins.

## Data Availability

The dataset used and/or analyzed during the current study is available from the corresponding author on reasonable request.

## Abbreviations

CSVD: Cerebral small vessel disease
WMH: White matter hyperintensities
EPVS: Enlarged perivascular space
TR: Repetition time
TE: Echo time
FLAIR: Fluid-attenuated inversion recovery
ICV: Intracranial volume
HDL-C: High-density lipoprotein cholesterol
LDL-C: Low-density lipoprotein cholesterol
