# Supplementary material for: Assessing the effectiveness of statin therapy for alleviating cerebral small vessel disease progression in people ≥75 years of age
Source: BMC Geriatr. 2020 Aug 17;20:292. doi: 10.1186/s12877-020-01682-w (PMC7430010; doi:10.1186/s12877-020-01682-w)
Supplement: Supplementary file 1 — Additional file 1: eTable 1. Demographic and clinical characteristics of the excluded participants ≥75 years of age in the cohort study. eTable 2. Demographic and clinical characteristics of the excluded hypertensive patients ≥75 years of age in the clinical trial. eTable 3. Mean and variability in blood pressure in the cohort study and clinical trial over the follow-up period. eTable 4. Adverse effects of statins. [file 12877_2020_1682_MOESM1_ESM.docx]

**Supplementary Material:**

**Assessing the effectiveness of statin therapy for alleviating cerebral small vessel disease progression in people ≥75 years of age**

**Short title: Statins and cerebral small vessel disease**

Yuqi Guo^1,2^,Yunpeng Li^3^, Xukui Liu^3^, Yi Cui^4^, Yingxin Zhao^1,5^, Shangwen Sun^1,5^, Qing Jia^1,5^, Qiang Chai^1,5^,Gary Gong^6^, Hua Zhang^1,5,*^, Zhendong Liu^1,5,*^

**Table S1. Demographic and clinical characteristics of the excluded participants ≥75 years of agein the cohort study**

|  | Non-statin group  (*n*=36) | Statin group  (*n*=10) | *P* value |
| --- | --- | --- | --- |
| Clinical parameters |  |  |  |
| Female (*n* [%]) | 18(50.0) | 6(60.0) | 0.575 |
| Age (years) | 77.53±2.05 | 78.20±2.57 | 0.390 |
| Current smoking (*n* [%]) | 13(36.1) | 3 (30.0) | 0.720 |
| Alcohol consumption (*n* [%]) | 10(27.8) | 5(50.0) | 0.185 |
| Hypertension (*n* [%]) | 23(63.9) | 7(70.0) | 0.720 |
| Antihypertensive medication (*n* [%]) | 17 (52.8) | 6(60.0) | 0.475 |
| Diabetes (*n* [%]) | 4(11.1) | 2(20.0) | 0.460 |
| Lowering glucose medication (*n* [%]) | 3 (8.3) | 2(20.0) | 0.294 |
| Dyslipidemia (*n* [%]) | 27(75.0) | 8(80.0) | 0.743 |
| Statins (*n* [%]) | 0 (0.0) | 10(100.0) | -- |
| Simvastatin | 0 (0.0) | 5 (50.0) | -- |
| Atorvastatin | 0 (0.0) | 11 (10.0) | -- |
| Lovastatin | 0 (0.0) | 0 (0.0) | -- |
| Fluvastatin | 0 (0.0) | 0 (0.0) | -- |
| Pravastatin | 0 (0.0) | 1 (10.0) | -- |
| Rosuvastatin | 0 (0.0) | 3 (30.0) | -- |
| Antiplatelet medication (*n* [%]) | 7(19.4) | 2 (20.0) | 0.969 |
| Body mass index (kg/m^2^) | 24.05±2.72 | 25.37±3.22 | 0.199 |
| Heart rate (bpm) | 71.69±7.99 | 75.50±7.18 | 0.181 |
| SBP (mm Hg) | 146.36±15.92 | 149.90±10.27 | 0.511 |
| DBP (mm Hg) | 71.00±10.85 | 80.20±7.18 | 0.015 |
| Biochemical parameters |  |  |  |
| TCHO (mmol/L) | 5.02±0.98 | 5.15±0.81 | 0.711 |
| TG (mmol/L) | 1.61±0.64 | 1.72±0.65 | 0.660 |
| HDL-C (mmol/L) | 1.32±0.47 | 1.16±0.33 | 0.334 |
| LDL-C (mmol/L) | 2.70±0.81 | 2.96±0.85 | 0.386 |
| FPG (mmol/L) | 5.72±1.12 | 5.93±1.26 | 0.611 |
| Brain magnetic resonance imaging |  |  |  |
| WMH (mL) | 5.22(4.44, 6.36) | 4.49(2.89, 6.09) | 0.053 |
| WMH-to-ICV ratio (%) | 0.41(0.31, 0.51) | 0.33(0.24, 0.42) | 0.076 |
| Prevalence of Fazekas scale ≥2 (*n* [%]) | 7(19.4) | 3(30.0) | 0.474 |
| Prevalence of lacunes (*n* [%]) | 4(11.1) | 1(10.0) | 0.920 |
| Prevalence of Virchow-Robin spaces (*n* [%]) | 6(16.7) | 2(20.0) | 0.806 |
| Prevalence of microbleeds (*n* [%]) | 4(11.1) | 1 (10.0) | 0.920 |

Data are expressed as mean ± standard deviation, median with interquartile range, or numbers with percentages. Abbreviation list: SBP, systolic blood pressure; DBP, diastolic blood pressure; TCHO, total cholesterol; TG, triglycerides; HDL-C, high-density lipoprotein cholesterol; LDL-C, low-density lipoprotein cholesterol; FPG, fasting plasma glucose; WMH, white matter hyperintensities; ICV, intracranial volume.

**Table S2. Demographic and clinical characteristics of the excluded hypertensive patients ≥75 years of age in the clinical trial**

|  | Placebo group  (*n*=15) | Rosuvastatin group  (*n*=9) | *P* value |
| --- | --- | --- | --- |
| Clinical parameters |  |  |  |
| Female (*n* [%]) | 2 (13.3) | 5 (55.6) | 0.028 |
| Age (years) | 79.73±3.06 | 78.11±1.69 | 0.160 |
| Current smoking (*n* [%]) | 8 (53.3) | 2 (22.2) | 0.134 |
| Alcohol consumption (*n* [%]) | 7(46.7) | 2(22.2) | 0.231 |
| Body mass index (kg/m^2^) | 23.47±2.10 | 23.54±2.32 | 0.941 |
| Heart rate (bpm) | 67.87±6.42 | 65.00±4.69 | 0.258 |
| SBP (mm Hg) | 158.07±7.91 | 159.11±11.66 | 0.796 |
| DBP (mm Hg) | 68.20±7.17 | 68.89±8.85 | 0.837 |
| Biochemical parameters |  |  |  |
| TCHO (mmol/L) | 5.26±0.64 | 5.00±0.77 | 0.385 |
| TG (mmol/L) | 1.37±0.31 | 1.22±0.28 | 0.259 |
| HDL-C (mmol/L) | 1.13±0.18 | 1.22±0.13 | 0.203 |
| LDL-C (mmol/L) | 3.51±0.67 | 3.23±0.72 | 0.337 |
| FPG (mmol/L) | 5.51±0.65 | 5.60±0.60 | 0.759 |
| Brain magnetic resonance imaging |  |  |  |
| WMH (mL) | 8.33(6.98, 9.06) | 6.64(4.62, 8.19) | 0.089 |
| WMH-to-ICV ratio (%) | 0.69(0.52, 0.74) | 0.57(0.36, 0.70) | 0.144 |
| Prevalence of Fazekas scale ≥2 (*n* [%]) | 3(20.0) | 2(22.2) | 0.897 |
| Prevalence of lacunes (*n* [%]) | 2(13.3) | 1 (11.1) | 0.873 |
| Prevalence of Virchow-Robin spaces (*n* [%]) | 3(20.0) | 1 (11.1) | 0.572 |
| Prevalence of microbleeds (*n* [%]) | 1(6.7) | 0 (0.00) | 0.429 |

Data are expressed as mean ± standard deviation, median with interquartile range, or numbers with percentages. Abbreviation list: SBP, systolic blood pressure; DBP, diastolic blood pressure; TCHO, total cholesterol; TG, triglycerides; HDL-C, high-density lipoprotein cholesterol; LDL-C, low-density lipoprotein cholesterol; FPG, fasting plasma glucose; WMH, white matter hyperintensities; ICV, intracranial volume.

**Table S3. Mean and variability in blood pressure in the cohort study and clinical trial over the follow-up period**

| A. The cohort study |  |  |  |
| --- | --- | --- | --- |
|  | Non-statin group  (*n*=698) | Statin group  (*n*=129) | *P* value |
| Mean SBP (mm Hg) | 146.34±23.81 | 146.76±24.39 | 0.740 |
| Mean DBP (mm Hg) | 72.55±11.42 | 73.18±12.56 | 0.571 |
| SD of SBP (mm Hg) | 19.83±11.74 | 17.79±12.31 | 0.072 |
| SD of DBP (mm Hg) | 9.64±5.17 | 8.49±4.83 | 0.019 |
| 1. The clinical trial study |  |  |  |
|  | Placebo group  (*n*=109) | Rosuvastatin group  (*n*=118) | *P* value |
| Mean SBP (mm Hg) | 143.27±20.33 | 142.48±18.96 | 0.762 |
| Mean DBP (mm Hg) | 66.93±5.71 | 66.87±6.16 | 0.939 |
| SD of SBP (mm Hg) | 14.26±9.77 | 12.54±9.23 | 0.174 |
| SD of DBP (mm Hg) | 6.81±4.33 | 6.15±4.54 | 0.285 |

Data are expressed as mean ± standard deviation. Abbreviation list: SBP, systolic blood pressure; DBP, diastolic blood pressure; SD, standard deviation.

**Table S4. Adverse effects of statins**

| A. The cohort study |  |  |  |
| --- | --- | --- | --- |
|  | Non-statin group  (*n*=698) | Statin group  (*n*=129) | *P* value |
| Incident new diagnosed diabetes (*n* [%]) | 49 (7.0) | 11 (8.5) | 0.544 |
| Myalgias (*n* [%]) | 5 (0.7) | 3 (2.3) | 0.086 |
| Impaired liver function (*n* [%]) | 23 (3.3) | 8 (6.2) | 0.110 |
| Frailty (*n* [%]) | 46 (6.6) | 9 (7.0) | 0.871 |
| 1. The clinical trial study |  |  |  |
|  | Placebo group  (*n*=109) | Rosuvastatin group  (*n*=118) | *P* value |
| Incident new diagnosed diabetes (*n* [%]) | 8 (7.3) | 11 (9.3) | 0.590 |
| Myalgias (*n* [%]) | 1 (0.9) | 3 (2.5) | 0.353 |
| Impaired liver function (*n* [%]) | 3 (2.8) | 7 (5.9) | 0.243 |
| Frailty (*n* [%]) | 3 (2.8) | 4 (3.4) | 0.781 |
